# Caesarean section and anal incontinence in women after obstetric anal sphincter injury: A systematic review and meta‐analysis

**DOI:** 10.1111/1471-0528.17899

**Published:** 2024-07-04

**Authors:** Emily Carter, Rebecca Hall, Kelechi Ajoku, Jenny Myers, Rohna Kearney

**Affiliations:** ^1^ The Warrell Unit, Saint Mary's Hospital, Manchester University NHS Foundation Trust Manchester Academic Health Science Centre Manchester UK; ^2^ Division of Developmental Biology and Medicine, School of Medical Sciences, Faculty of Biology, Medicine and Health University of Manchester Manchester UK; ^3^ Maternal and Fetal Health Research Centre, Saint Mary's Hospital, Manchester University NHS Foundation Trust, Manchester Academic Health Science Centre University of Manchester Manchester UK

**Keywords:** anal incontinence, caesarean, faecal incontinence, mode of birth, OASIS, obstetric anal sphincter injury, planned birth, quality of life, recurrent OASI, shared decision‐making, vaginal birth

## Abstract

**Background:**

Approximately 50% women who give birth after obstetric anal sphincter injury (OASI) develop anal incontinence (AI) over their lifetime.

**Objective:**

To evaluate current evidence for a protective benefit of planned caesarean section (CS) to prevent AI after OASI.

**Search Strategy:**

MEDLINE/PubMed, Embase 1974–2024, CINAHL and Cochrane to 7 February 2024 (PROSPERO CRD42022372442).

**Selection Criteria:**

All studies reporting outcomes after OASI and a subsequent birth, by any mode.

**Data Collection and Analysis:**

Eighty‐six of 2646 screened studies met inclusion criteria, with nine studies suitable to meta‐analyse the primary outcome of ‘adjusted AI’ after OASI and subsequent birth. Subgroups: short‐term AI, long‐term AI, AI in asymptomatic women. Secondary outcomes: total AI, quality of life, satisfaction/regret, solid/liquid/flatal incontinence, faecal urgency, AI in women with and without subsequent birth, change in AI pre‐ to post‐ subsequent birth.

**Main Results:**

There was no evidence of a difference in adjusted AI after subsequent vaginal birth compared with CS after OASI across all time periods (OR = 0.92, 95% CI 0.72–1.20; 9 studies, 2104 participants, *I* ^2^ = 0% *p* = 0.58), for subgroup analyses or secondary outcomes. There was no evidence of a difference in AI in women with or without subsequent birth (OR = 1.00 95% CI 0.65–1.54; 10 studies, 970 participants, *I* ^2^ = 35% *p* = 0.99), or pre‐ to post‐ subsequent birth (OR = 0.79 95% CI 0.51–1.25; 13 studies, 5496 participants, *I* ^2^ = 73% *p* = 0.31).

**Conclusions:**

Due to low evidence quality, we are unable to determine whether planned caesarean is protective against AI after OASI. Higher quality evidence is required to guide personalised decision‐making for asymptomatic women and to determine the effect of subsequent birth mode on long‐term AI outcomes.

## 1 INTRODUCTION

Anal incontinence (AI), (the involuntary passage of solid/liquid stool or flatus), and faecal incontinence, (the involuntary passage of solid or liquid stool), are life‐changing complications of obstetric anal sphincter injury (OASI).^1^ OASI affects 6% primiparous births—approximately 10 000 UK women per year.^2^, ^3^ The incidence of OASI in primiparous women tripled to 5.9% in the United Kingdom between 2000 and 2012.^3^ The incidence of AI is three times higher in women after OASI than women without; this rises proportionate to degree of injury.^4^, ^5^

OASI most commonly affects primiparous women; approximately 75% have further children.^6^, ^7^ Ten per cent of women who are initially asymptomatic of AI after OASI become symptomatic by 3 years.^8^ Half of women (range 38.9%–64.5%) who have an OASI and another vaginal birth (VB) develop AI over their lifetime.^9^, ^10^, ^11^, ^12^, ^13^, ^14^, ^15^ Women who have recurrent OASI have a 50.0% incidence of AI by 8.5 years and a 48.8% incidence of faecal incontinence by the age of 60 years.^12^, ^16^ Pre‐existing symptoms (‘transient’ or ‘permanent’ AI after index birth), correlate with long‐term AI regardless of any subsequent birth, or birth mode.^11^, ^17^ In women with an OASI in their first birth and one subsequent vaginal birth, the long‐term incidence of AI is 58.8% after a fourth‐degree tear and 41.0% after a third‐degree tear.^10^

The ‘OASIS syndrome’ describes the effect of AI on quality of life (QOL) and includes feeling unclean, isolated, mutilated, grieving and negatively impacts motherhood, sexual intimacy and partner relations.^18^ QOL is inversely proportional to AI symptoms and number of OASI births.^19^, ^20^ Studies investigating QOL in women with AI after OASI demonstrate that even small differences in AI (2 on a 24‐point Vaizey incontinence scale) have a significant impact for women.^19^, ^21^

Based on expert opinion, the Royal College of Obstetricians and Gynaecologists recommends that caesarean birth is discussed with women with AI symptoms and those with abnormal endoanal ultrasonography and/or manometry after OASI. Specialised perineal clinics that facilitate mode of birth discussion are associated with high patient satisfaction.^22^ Concerns revolve around developing new or worse AI, which profoundly affects QOL.^18^, ^19^, ^21^, ^23^, ^24^ Due to low evidence quality for recommendations, protocols vary extensively; between 10%–83.6% women were advised to deliver by caesarean after OASI in published studies.^7^, ^9^, ^20^, ^25^, ^26^, ^27^, ^28^, ^29^, ^30^ For the majority of women, mode of birth discussions are based on women's symptoms and preferences alone due to limited availiability of anorectal investigations.^31^

### 1.1 Objective

The objectives of this study were as follows.

1. To evaluate current evidence for whether subsequent birth impacts development of anal incontinence after OASI.
2. To determine whether subsequent Caesarean section (CS) is protective against the development of anal incontinence compared with subsequent vaginal birth (VB).

## 2 METHODS

This systematic review and meta‐analysis was performed according to prospectively published methodology (PROSPERO CRD42022372442) using PRISMA guidelines.^32^ Study authors were contacted to provide data in suitable format for meta‐analysis if required. Core outcome sets for this topic have not been published; however protocols are in development for OASI,^33^ perineal trauma research^34^ and the effect of episiotomy at birth on OASI.^35^ Patients and the public were not directly involved in conducting this review; results and implications have been discussed with an advisory group of affected women. This study was not externally funded.

### 2.1 Eligibility criteria

#### 2.1.1 Participants/population

##### Inclusion criteria

Studies including women who have had an OASI and a subsequent birth, by any mode. OASI is defined as a third‐ or fourth‐degree tear by the Sultan classification.^5^ Studies were included if injury to the anal muscle, canal or mucosa was described, and descriptive studies relating to outcome(s) of interest.

##### Exclusion criteria

Studies not clearly documenting OASI or an equivalent population, case series/case reports and retrospective audits, which do not contain outcome(s) of interest or do not describe outcomes after subsequent birth.

#### 2.1.2 Intervention

Planned birth by CS after an OASI analysed by intention to treat if available.

#### 2.1.3 Comparison

Planned VB. Emergency CS data were analysed with VB outcomes where possible. Observational studies comparing VB to all CS (emergency and elective) using retrospective data were included, with risk of bias assessed accordingly.

#### 2.1.4 Outcomes

##### Primary outcome

‘Adjusted AI’ after subsequent birth, including (but not limited to): St Mark's Score, Wexner/Vaizey score, EPAQ and clinical description. ‘Adjusted AI’ is defined as either^1^ any ‘new’ or ‘worsening’ AI symptoms, or^2^ descriptive development of AI symptoms in a previously asymptomatic woman after a subsequent birth after OASI.

##### Subgroup analysis

Short‐term AI (≤2 year), long‐term AI (≥5 year), AI development in women asymptomatic after index birth.

##### Secondary outcomes

‘Total AI’ (unadjusted total incidence of AI after a subsequent OASI birth), incontinence of solid stool, liquid stool, flatus, and faecal urgency symptoms, QOL, regret, satisfaction with mode of birth choice, obstructive defecation, change in AI pre‐ and post‐subsequent birth, AI in women after subsequent birth versus no subsequent birth, repeat OASI rate and adverse events (return to theatre, organ injury).

### 2.2 Search strategy

MeSH subject heading and database‐specific truncated search terms were used to avoid excluding potential studies; our search strategy is detailed in Appendix S1. Three concepts were implemented: (1) identification of all types of anal sphincter or anal canal injury, (2) identification of subsequent birth(s) by any mode and (3) identification of all types of anal or faecal incontinence.

### 2.3 Information sources

Search strategies were executed in Ovid MEDLINE/PubMed 1946–2024 (Ovid), Embase 1974–2024 (Ovid), CINAHL (EBSCOhost) 1937–2024, Cochrane Combined 1996–2024, Clinical trials and Google Scholar from inception to 07 February 2024.

### 2.4 Study selection

#### 2.4.1 Setting

**FIGURE 1:**
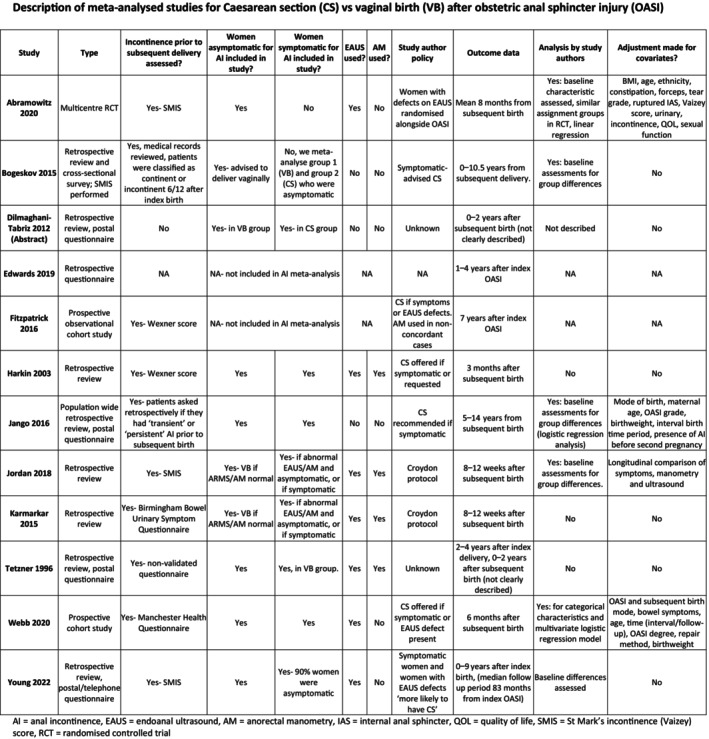
Characteristics of included studies.

We included randomised controlled trials (RCTs) and non‐randomised studies including observational, cohort, cross‐sectional, case–control and retrospective reviews, in any publication format that provided outcome data for any birth after OASI. Studies were meta‐analysed if theyprovided data for one of three comparisons: (1) after a subsequent CS compared with subsequent VB, (2) if they provided AI data for a subsequent birth by any mode compared with no subsequent birth or (3) if they provided paired AI data for pre‐ versus post‐subsequent birth. Characteristics of the meta‐analysed studies are described for our primary AI outcome of subsequent CS versus subsequent VB (Figure 1).

### 2.5 Data extraction (selection and coding)

All title/abstract screening (EC and RH), full‐text review (EC and RH), data extraction (EC, KA and RH) and risk‐of‐bias assessments (EC and RH) were completed by two blinded independent reviewers according to standard Cochrane methodology. Screening took place using the Rayyan review software.^36^ Discrepancies were resolved by a third reviewer (RK). We excluded publications describing the same participants.^29^, ^37^

### 2.6 Risk‐of‐bias assessment

We used the Risk Of Bias In Non‐randomised Studies of Interventions (ROBINS‐I) tool as per the Cochrane handbook to assess risk of bias in included studies.^38^, ^39^

### 2.7 Strategy for data synthesis

Continuous variables (e.g. AI on incontinence score) were converted to dichotomous outcomes for meta‐analysis in RevMan 5.4. Data are presented as odds ratios with 95% confidence intervals (CIs). When significant statistical heterogeneity was demonstrated on meta‐analysis (including, but not limited to *I* ^2^ > 40%), random effect modelling is used.

### 2.8 Subgroup analysis

Performed for primary outcome as per protocol at pre‐specified time points and for women asymptomatic of AI after OASI.

## 3 RESULTS

### 3.1 Overview

**FIGURE 2:**
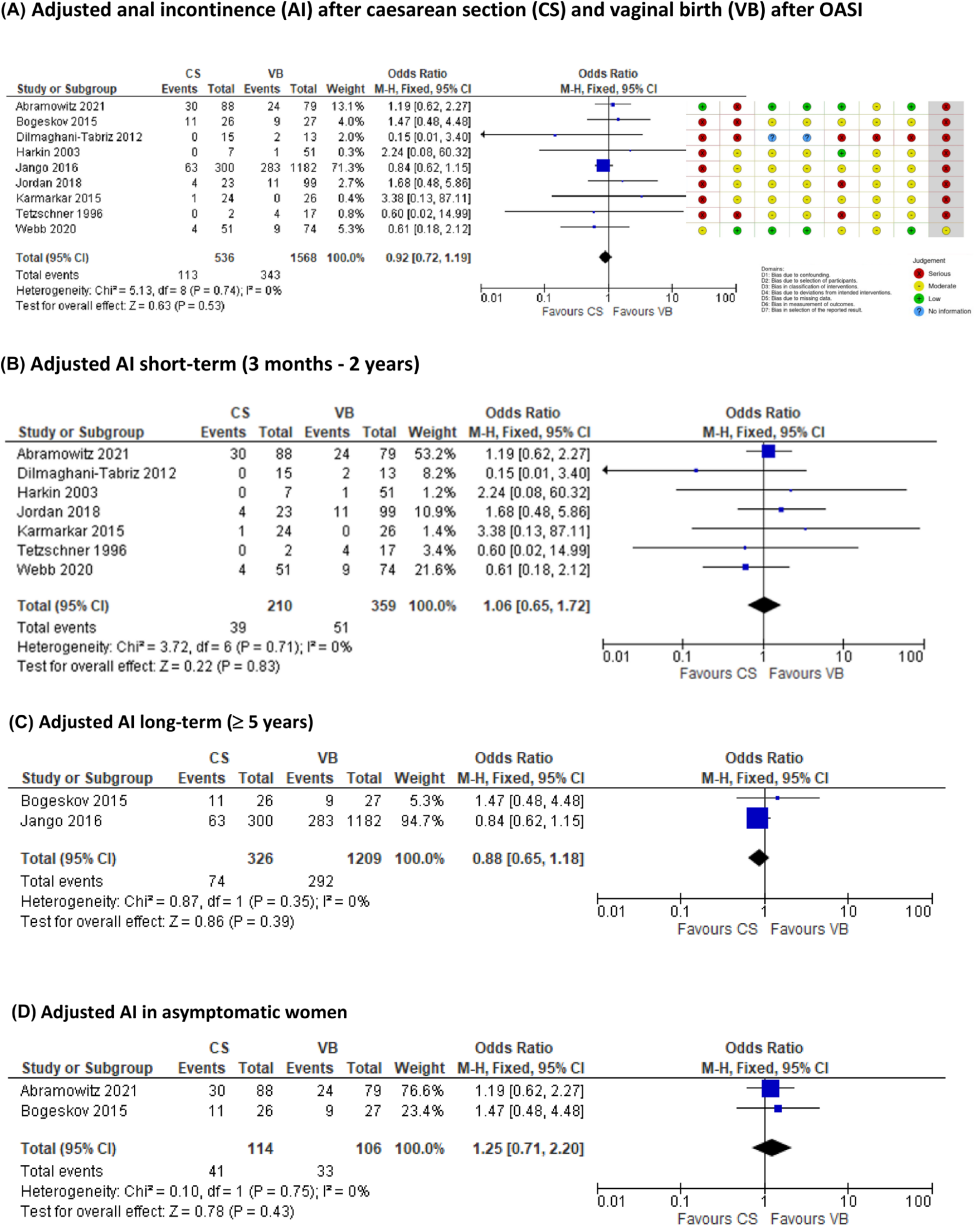
Adjusted anal incontinence (AI) after caesarean section (CS) and vaginal birth (VB) after obstetric anal sphincter injury, risk of bias in non‐randomised studies of interventions, and subgroup analyses.

**FIGURE 3:**
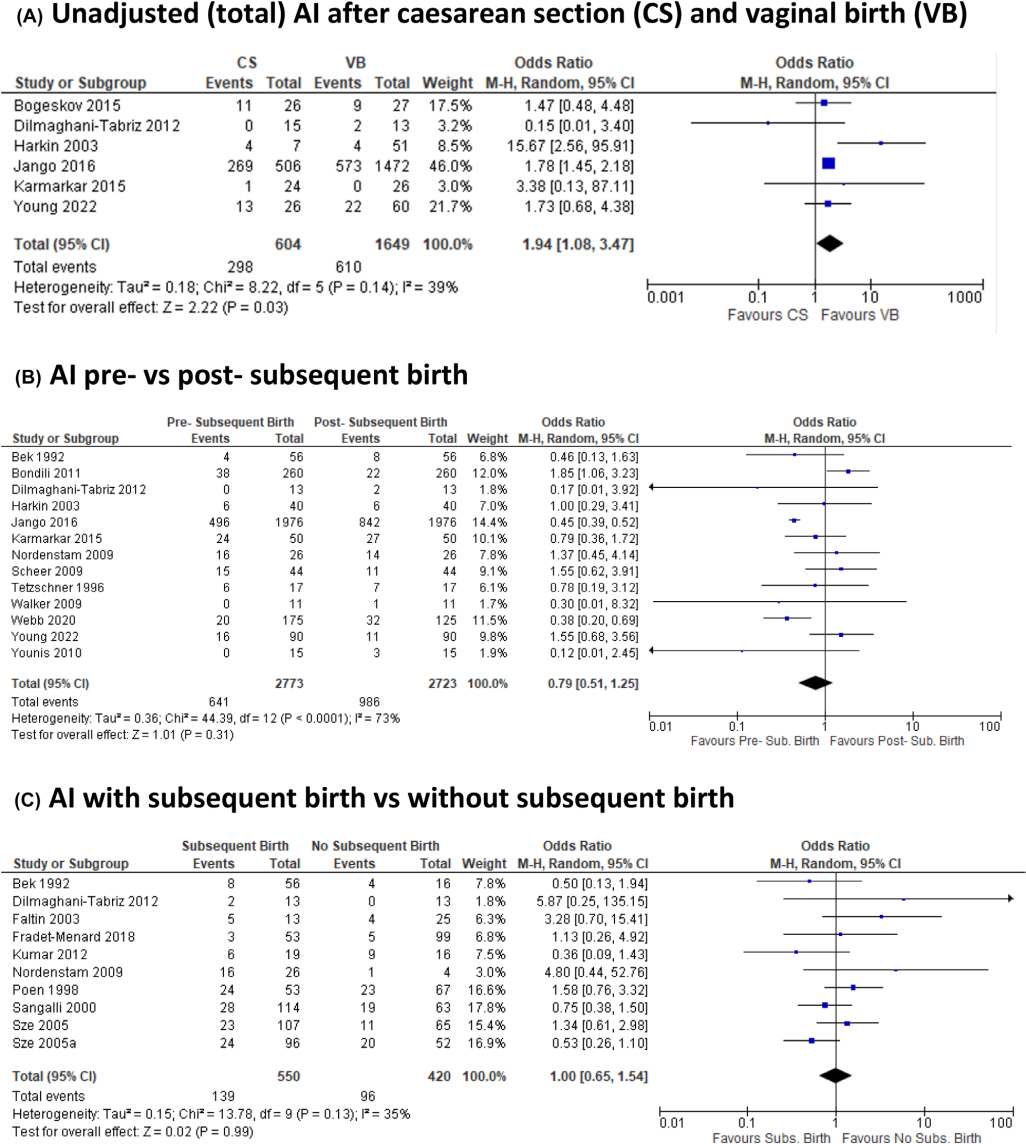
Secondary outcomes for anal incontinence (AI) after obstetric anal sphincter injury. A: Unadjusted (total) incidence of AI after VB and CS. B: Difference in AI before and after a subsequent birth. C: AI in women with and without subsequent birth.

A total of 2646 studies were identified. Eighty‐six studies met broad inclusion criteria after full‐text review (PRISMA diagram, Figure S1). Twelve studies were included in meta‐analysis for an AI outcome as they provided data for both CS and VB. Nine studies were included in our primary outcome ‘adjusted AI’ (Figure 2). One study did not provide an adjusted value and is included only in ‘Total AI’ (Figure 3).^40^ Two studies provided data for ‘satisfaction’ and ‘regret’ but not for AI.^7^, ^41^

**FIGURE 4:**
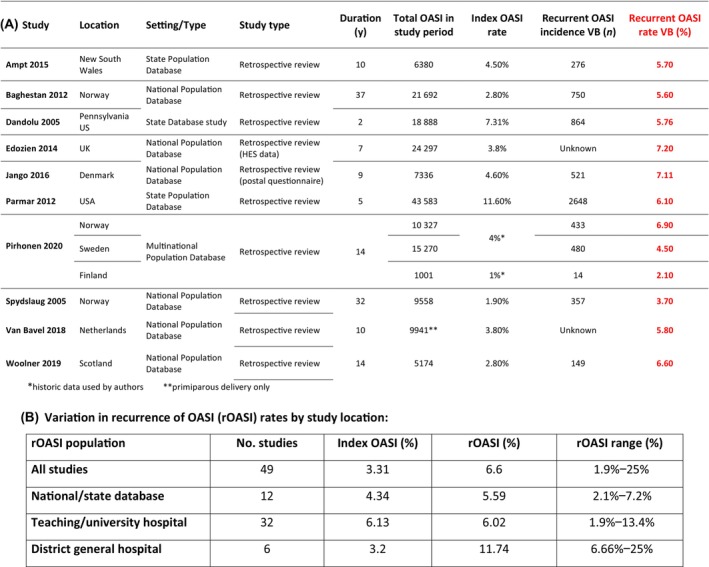
Summary of index and recurrent obstetric anal sphincter injury rates in (A) populaton databases and (B) variation by study location.

Ten studies contributed to meta‐analysis for AI after subsequent birth by any mode after OASI (Figure 3). Thirteen studies contributed to meta‐analysis for AI pre‐ versus post‐subsequent birth by any mode after OASI (Figure 3). Forty‐nine studies provided data values for the incidence of recurrent OASI in a subsequent VB after OASI (Figure 4 and Table S1).

### 3.2 Characteristics of included studies

Studies varied in quality and methodology. For the 12 studies meta‐analysed for outcomes after a VB and CS, 1 RCT and 11 non‐randomised studies were included. The non‐randomised studies included retrospective reviews, case–control studies, service evaluations and questionnaire studies (Figure 1). Eight studies used validated assessment methods.^7^, ^11^, ^20^, ^27^, ^30^, ^41^, ^42^, ^43^ Four studies used unvalidated or descriptive assessments.^27^, ^44^, ^45^, ^46^ Two studies provided extractable data for women who were asymptomatic of AI after the index OASI (Figure 2).^42^, ^43^

### 3.3 Risk of bias

The ROBINS‐I tool demonstrated that eight out of nine studies contributing data to the meta‐analysis for ‘adjusted AI’ were at high risk of bias for at least one domain (Figure 1).^39^ Non‐randomised studies including women who were symptomatic of AI after OASI were at risk of confounding in allocation to CS and VB groups.

One RCT contributed data to meta‐analysis. 22.1% of 222 women randomised had a third‐degree tear diagnosed at the time of the index birth; the other participants were recruited after a forceps birth and had an anal sphincter defect identified on endoanal ultrasound, which was considered a surrogate marker for OASI.^43^

One prospective cohort study with a six month follow‐up using a valid AI symptom score was included.^20^ All other studies were deemed high risk of bias by the ROBINS‐I tool. These were limited by: confounding by inclusion of women symptomatic of AI, retrospective recording, not accounting for important baseline variables such as parity and performing retrospective analyses, for instance analysing all CS data together (emergency/elective), or omitting emergency CS data.

Studies providing longer term follow‐up data were deemed high risk of bias by the ROBINS‐I tool. These were limited by: retrospective recall of symptoms (e.g. years after birth), variations in included populations (e.g. patients with OASI diagnosed sonographically, exclusion of women with OASI recurrence and exclusion of emergency CS), use of non‐validated symptom assessment tools, selective outcome reporting, use of multiple analyses and methodological limitations such as retrospective service evaluation, limited follow‐up response rate and directive counselling for mode of birth.

### 3.4 Data heterogeneity

A wide range of methods and protocols for counselling mode of subsequent birth were described based on patient's symptoms alone, endoanal ultrasound, anorectal manometry and findings on 3D transperineal ultrasound. Seven studies used endoanal ultrasound to counsel women regarding mode of subsequent birth^27^, ^30^, ^40^, ^43^, ^45^, ^46^, ^47^ and four used anorectal manometry^27^, ^30^, ^45^, ^46^ (Figure 1). One study used 3D‐transperineal ultrasound but did not contribute data to meta‐analysis.^9^

### 3.5 Primary outcome: Adjusted anal incontinence (AI) after subsequent birth (Figure 2A)

Nine studies (including one RCT) were suitable for meta‐analysis; their characteristics are described (Figure 1). Eleven studies were not suitable for meta‐analysis due to either a lack of both CS and VB groups or they did not provide individual data for AI (either in publication or via contacting study authors).^7^, ^9^, ^15^, ^17^, ^48^, ^49^, ^50^, ^51^, ^52^, ^53^, ^54^

There was no evidence of a difference in adjusted AI (new or worsening AI symptoms) after subsequent VB compared with CS after OASI across all follow‐up time periods studied (OR = 0.92, 95% CI 0.72–1.20; 9 studies, 2104 participants, *I* ^2^ = 0%, *p* = 0.58) (Figure 2). One large population‐based study contributed significantly to meta‐analysis.^11^

Two studies that were not suitable for meta‐analysis demonstrated mixed results. The first study demonstrated significantly lower Vaizey score in women after CS than in women after VB; those following author's mode of birth recommendations also demonstrated significantly less AI.^52^ Although Wexner scores were higher in women delivering by CS, there was no difference in the average pre‐ and post‐birth scores in the CS and VB groups in another study.^7^

### 3.6 Subgroup analysis

#### 3.6.1 Short‐term AI ≤2 year (Figure 2B)

There was no evidence of a difference for short‐term symptoms at 3–24 months after birth (OR = 1.09, CI 0.67–1.80, 7 studies, 569 participants, *I* ^2^ = 0%, *p* = 0.72).

#### 3.6.2 Long‐term AI ≥5 year (Figure 2C)

There was no evidence of a difference in symptoms at ≥5 years (OR = 0.88, CI 0.65–1.18, 2 studies, 1535 participants, *I* ^2^ = 0%, *p* = 0.39).

#### 3.6.3 Asymptomatic women (Figure 2D)

There was no evidence of a difference in AI symptoms in women who delivered subsequently by VB and CS in studies who recruited only asymptomatic women (OR = 1.25, CI 0.71–2.20, 2 studies, *I* ^2^ = 0%, 220 participants *p* = 0.43).

### 3.7 Secondary outcomes

#### 3.7.1 Total AI—Unadjusted (Figure 3A)

The total incidence of AI after subsequent birth after OASI may be higher after CS than VB; the wide CI is consistent with little effect or harm (OR 1.94, 95% CI 1.08–3.47; 6 studies, 2253 participants, *I* ^2^ = 39%, *p* = 0.03). These studies include women who were symptomatic pre‐subsequent birth. Symptomatic women were more likely to have CS than VB and to remain symptomatic after subsequent birth in the short and long term.

#### 3.7.2 Incontinence of solid stool, liquid stool, flatus and faecal urgency (Figure S2)

The incidence of incontinence of solid or liquid stool, flatal incontinence and faecal urgency does not differ between women who have a subsequent CS versus VB (Figure S2).

#### 3.7.3 Quality‐of‐life (QOL) related to birth mode (Figure S3)

There was no evidence of a difference in QOL related to birth mode on meta‐analysis (Figure S3). Two studies described QOL after a subsequent birth by VB and CS after OASI.^11^, ^20^ One study demonstrated higher levels of ‘incontinence impact’ and ‘physical limitations’ in women having CS versus VB.^20^ Reduction in QOL is directly proportional to patient symptoms of AI after OASI.^29^

#### 3.7.4 Regret and satisfaction with birth mode (Figure S3)

There is no evidence of a difference in levels of satisfaction^7^, ^40^ and regret with subsequent CS compared with subsequent VB after OASI^41^, ^42^ (Figure S3); regret may be inversely proportional to satisfaction.^41^

#### 3.7.5 Change in AI pre‐ and post‐subsequent birth (Figure 3B)

The incidence of AI pre‐ and post‐subsequent birth does not differ across all time periods studied (OR = 0.79, CI 0.51–1.25, 13 studies, 5496 participants, *I* ^2^ = 76%, *p* = 0.31).^11^, ^17^, ^20^, ^27^, ^29^, ^40^, ^44^, ^45^, ^46^, ^53^, ^55^, ^56^, ^57^ These data include the same women pre‐ and post‐subsequent birth by any mode after OASI, and represent a mixture of symptommatic and asymptommatic women (Figure 3).

#### 3.7.6 AI after subsequent birth versus no subsequent birth (Figure 3C)

Twelve studies have investigated a difference in AI in women who have a subsequent birth compared with women who have not had a subsequent birth after OASI. Ten studies were suitable for meta‐analysis.^17^, ^44^, ^51^, ^56^, ^58^, ^59^, ^60^, ^61^, ^62^, ^63^ There is no evidence of a difference in AI in women who have a subsequent birth after OASI by any mode versus those who have not (OR = 1.00, 95% CI 0.65–1.54; 10 studies 970 participants, *I* ^2^ = 35%, *p* = 0.99) (Figure 3).

One study looked at QOL in women with and without subsequent birth (Figure S3). There was no difference in QOL outcomes for women with a subsequent birth by any mode compared with those who did not.^58^ A previous review meta‐analysed this outcome in 2016.^47^ Only one further study has been added since this time.^20^

#### 3.7.7 Recurrence of OASI in subsequent VB (Figure 4 and Table S1)

Forty‐nine studies reported incidence of OASI recurrence in women undergoing subsequent VB (Table S1). These included 10 national or state database studies,^6^, ^11^, ^12^, ^64^, ^65^, ^66^, ^67^, ^68^, ^69^, ^70^, ^71^ 3 US state databases^6^, ^65^, ^67^ and 1 Canadian state database (Figure 4).^71^ Three studies were multicentre studies^42^, ^43^, ^72^ and 32 studies were single‐centre studies.^7^, ^9^, ^15^, ^30^, ^40^, ^41^, ^44^, ^45^, ^46^, ^48^, ^49^, ^52^, ^54^, ^58^, ^73^, ^74^, ^75^, ^76^, ^77^, ^78^, ^79^, ^80^, ^81^, ^82^, ^83^, ^84^, ^85^, ^86^, ^87^, ^88^, ^89^, ^90^ Twenty‐nine studies took place in a university or teaching hospital setting,^7^, ^9^, ^15^, ^40^, ^41^, ^42^, ^43^, ^45^, ^46^, ^48^, ^49^, ^52^, ^54^, ^58^, ^72^, ^74^, ^76^, ^77^, ^78^, ^79^, ^80^, ^81^, ^82^, ^83^, ^84^, ^85^, ^86^, ^87^, ^90^ and six were in a district general hospital (DGH) setting.^30^, ^44^, ^73^, ^75^, ^88^, ^89^

The mean index OASI rate was 3.31%, and mean recurrence rate was 6.6% across all studies (7413 cases: range 1.9%–25%, (Figure 4). Index OASI rates were higher in teaching hospitals (6.13% *n* = 32) than in population‐based studies (4.34% *n* = 11) and DGHs (3.2% *n* = 6). Recurrent OASI rates were higher in DGHs (11.74% range 6.6%–25% *n* = 6) but similar in teaching hospitals (6.02% range 1.9%–13.4% *n* = 32) and population‐based studies (5.59% range 2.1%–7.2% *n* = 11). A summary of recurrent OASI rates provided by population‐based studies (Figure 4) and all studies (Table S1) is described.

### 3.8 Other outcomes

No studies reported on symptoms of irritable bowel syndrome, obstructive defecation, blood loss or length of stay.

## 4 DISCUSSION

### 4.1 Main findings

#### 4.1.1 The preventative value of caesarean

This analysis does not demonstrate a protective benefit for routine elective CS in preventing new or worsening symptoms of AI after OASI in a group of women who are both symptomatic and asymptomatic after their index OASI. This was analysed from either a deterioration on a validated scoring system or a description of new or worsening symptoms in women by study authors after a subsequent birth after OASI. There is no evidence of difference in deterioration of AI between women who deliver by CS compared with women who deliver by VB after OASI overall, in the shorter term or longer term (Figure 2B‐C). For two studies in which an asymptomatic group was analysed, there was no evidence of a difference in AI symptoms in the short term; both studies are at high risk of bias (Figure 2D).

Regarding evidence certainty, 11 of the 12 studies providing data for AI outcome(s) were at high risk of bias for at least one domain utilising the ROBINS‐I tool (Figure 2A). This was due to limitations with the population recruited; non‐randomisation to treatment outcomes; inclusion of symptomatic women; retrospective analysis; failure to analyse by intention‐to‐treat (emergency CS group included with elective CS group or excluded); and lack of validated outcome data measurement. Meta‐analysis for our primary outcome was dominated by one large retrospective study.^11^ Overall, these limitations make clinical interpretation difficult; we do not have high‐quality data available to counsel women with and without symptoms of AI of their risk.

#### 4.1.2 Long‐term data

Long‐term data are determined almost exclusively from one large population‐based study.^11^ Women subsequently delivering by VB after OASI may experience some worsening of AI symptoms 5–12 years after subsequent birth compared with CS; however, this was not significant on the author's multivariate analysis.^11^ The other study providing long‐term data outcomes compared two different control groups of 50 patients and was not powered to investigate long‐term AI symptoms.^42^ We need higher quality data to investigate differences in long‐term AI symptoms, specifically for asymptomatic women, who deliver by VB and CS after OASI.

#### 4.1.3 Recurrence of OASI

Women who sustain OASI recurrence have poorer long‐term incontinence outcomes than women who deliver vaginally without another OASI.^10^ Rates of index OASI are higher in teaching and university hospitals than in population‐based studies and DGHs. This may be due to differences in OASI detection. Rates of recurrent OASI are highest in DGHs, and there is a 25‐fold difference in OASI recurrence rates reported across studies. OASI can be prevented via ongoing national initiatives.^91^, ^92^

### 4.2 Strengths and limitations

This comprehensive review summarises both current preventative evidence of CS for AI in women after OASI and the influence of subsequent birth on AI after OASI. It uses a robust prospectively published systematic methodology to double‐extract data and appraise risk of bias using the ROBINS‐I tool by two independent study authors. This is an extremely important clinical area, as AI has a profound negative effect on the lives of young women, OASI is common, and AI is difficult to manage in this cohort.^18^ This area is under‐researched; current guidelines utilise Level 4 evidence, and there is consequently widespread variation in clinical practice.^3^, ^31^

This study meta‐analyses incidence of new or worsening AI after subsequent VB compared with subsequent CS after OASI. It also analyses total incidence of AI after subsequent VB compared with subsequent CS after OASI. To our knowledge, it is the first study to investigate both outcomes. It highlights challenges appraising evidence from non‐randomised studies due to significant confounding caused by including women with pre‐existing symptoms of AI, and the need for higher‐quality data. Advising women of the preventative value of CS for AI after OASI is therefore complex as women often want personalised information in relation to thier symptom ptofile.

#### 4.2.1 Quality of the data

The vast majority of included studies were at high risk of bias on the ROBINS‐I tool (Figure 2). All studies except two included women with pre‐existing AI symptoms. Most studies are service evaluations. Validated AI scores are not used by the majority of studies to pair data pre‐ and post‐birth after OASI. Data quality is therefore significantly limited by confounding.

#### 4.2.2 Data heterogeneity

The use of endoanal ultrasound, anorectal manometry and transperineal scanning to assess anal sphincter defects varied (Figure 1). Defects on EAUS are significantly correlated with symptoms of AI both in women with and without an OASI diagnosis.^93^ We include all women in meta‐analyses; however, it would be valuable to appreciate the long‐term value of anorectal investigations in preventing AI in asymptomatic women who undergo subsequent birth after OASI.

#### 4.2.3 Deviations from study protocol

It was not possible to stratify AI by our pre‐specified follow‐up period due to ranges employed by particular studies (Figure S2).^11^, ^40^, ^44^, ^46^ We adopted a pragmatic approach and subgrouped data at 3–24 months (short term) and ≥5 year (long term).

### 4.3 Interpretation of results

This systematic review has not demonstrated a protective effect of CS over VB to prevent new or worsening AI symptoms after OASI . Meta‐analysed data are low‐quality and represent a mixed group of women with and without symptoms of AI after index OASI birth.^7^, ^27^, ^29^, ^30^, ^44^, ^45^, ^57^, ^94^ Nearly all studies were of short follow‐up duration. It therefore does not reliably assess an effect on long‐term AI outcomes or provide an individualised risk assessment for women who are symptomatic or asymptomatic after OASI. Evidence to support mode of birth counselling regarding risk of AI after OASI is therefore limited.

There is a paucity of data on women's preferences and decision‐making.^95^ Factors predicting worse AI with subsequent birth after OASI include transient or ongoing AI symptoms after index birth,^17^ fourth‐degree tear^5^, ^10^ and recurrent OASI.^12^ Factors predicting better AI outcomes include the absence of AI after index birth,^17^ normal anorectal physiology,^30^ lesser degree of injury^5^ and absence of injury at subsequent birth.^12^ Future work should concentrate on addressing evidence gaps in using existing information to provide personalised risk information to women.

## 5 CONCLUSION

This systematic review and meta‐analysis is, to our knowledge, the only high‐quality summary of AI outcomes in women who give birth after OASI. Evidence is low‐quality, and it is difficult to draw clinically meaningful conclusions. Current studies do not support the routine practice of elective caesarean to prevent new or worsening AI symptoms; however, they are based on a mixed group of women both with and without AI, and mostly report outcomes in the shorter term.

There are significant limitations, specifically relating to the clinically important group of women who are asymptomatic after OASI and long‐term AI outcomes. The publication of future high‐quality studies with long‐term follow‐up should include asymptomatic women with and without anal sphincter defects. This required to provide risk information for personalisation of birth choice.

### 5.1 Practical and research recommendations

In the United Kingdom, the average woman sustaining an OASI with her first birth can expect to live with any AI symptoms for 50 years. There is a 36.8% rate of faecal incontinence in women with one OASI aged over 40 years.^16^ Support for birth choice is prioritised by the NHS 3‐year maternity and long‐term plans.^96^, ^97^ Higher quality evidence is required to provide meaningful data on the risk of future birth mode on AI after OASI and facilitate informed birth choice. This should differentiate risk for women asymptomatic and symptomatic of AI. Research to ascertain the value of anorectal physiology investigations to predict longer term AI would be beneficial and enable standardisation of antenatal counselling pathways.

## AUTHOR CONTRIBUTIONS

EC was involved in project design and conception, prospectively registered the study and independently performed all aspects of the systematic review and meta‐analysis including title/abstract screening, data extraction, data analysis, risk of bias assessments, statistical analysis, interpretation of results and project write‐up. RH performed independent title/abstract screening, independent double data extraction for the recurrence of OASI data and independent risk‐of‐bias assessments. KA performed independent double‐data extraction for the included studies. JM contributed substantially to write‐up and clinical interpretation of results. RK was instrumental in project design and conception, performed data analysis and made substantial contributions to project write‐up and interpretation of results.

## CONFLICT OF INTEREST STATEMENT

The authors have no relevant financial, personal, political, intellectual or religious conflicts of interest to declare. This work was not externally funded.

## ETHICS APPROVAL

Ethics approval was not required for this study. The primary review author has completed systematic review author training with Cochrane UK.

## Supporting information


Appendix S1.

Figure S1.

Table S1.

Figure S2.

Figure S3.

## Data Availability

Data sharing is not applicable to this article as no new data were created or analyzed in this study.
