# Supplementary material for: Caesarean section and anal incontinence in women after obstetric anal sphincter injury: A systematic review and meta‐analysis
Source: BJOG. 2024 Jul 4;132(8):1032–44. doi: 10.1111/1471-0528.17899 (PMC12137769; doi:10.1111/1471-0528.17899)
Supplement: Supplementary file 1 — Appendix S1. [file BJO-132-1032-s004.docx]

**Search Strategy**

ID Search

#1 ((OASI or OASIS or obstetric anal sphincter injur*)):ti,ab,kw

#2 MeSH descriptor: [Anal Canal] explode all trees

#3 (((third or fourth) and degree tear*)):ti,ab,kw

#4 #1 OR #2 OR #3

#5 (mode of birth):ti,ab,kw

#6 (mode of delivery):ti,ab,kw

#7 ((vaginal and (birth or delivery))):ti,ab,kw

#8 (c?esarean*):ti,ab,kw

#9 MeSH descriptor: [Delivery, Obstetric] explode all trees

#10 #5 OR #6 OR #7 OR #8 OR #9

#11 ((Subsequent OR next) AND birth*):ti,ab,kw

#12 (Subsequent OR next) AND delivery*

#13 (Subsequent OR next) AND pregnanc*

#14 #12 OR #13

#15 #4 AND #10 AND #14

#16 #4 AND #10

We used #16 as it provided the broadest search
