# Supplementary material for: Caesarean section and anal incontinence in women after obstetric anal sphincter injury: A systematic review and meta‐analysis
Source: BJOG. 2024 Jul 4;132(8):1032–44. doi: 10.1111/1471-0528.17899 (PMC12137769; doi:10.1111/1471-0528.17899)
Supplement: Supplementary file 2 — Figure S1. [file BJO-132-1032-s005.docx]

**Identification of studies via other methods**

**Identification of studies via databases and registers**

Records identified from

Citation searching (n = 1)

Records removed *before screening*:

Duplicate records removed* (n = 1387)

Search Date 06/02/2024

Records identified from Medline/Pubmed 1209

Embase 2054

CINALH 553

Cochrane 15 + 202

(n = 4033)

**Identification**

Records screened (abstracts)

(n = 2646)

Records excluded**

(n = 2512)

Reports not retrieved

(n = 0)

Reports sought for retrieval

(n = 1)

Reports sought for retrieval

(n = 134)

Reports not retrieved

(n = 0)

**Screening**

Reports excluded:

Wrong study design 61

Wrong population 42

Wrong publication type 15

No/wrong outcome of interest 5

Duplicate population 11

Foreign language 0

Reports assessed for eligibility

(n = 1)

Reports excluded: 0

Reports assessed for eligibility (full text screening)

(n = 134)

Studies giving data for recurrent OASI incidence (n = 49)

Studies suitable for meta-analysis for AI outcome(s) after VB and CS (n = 12)***

Studies included in review

(n = 86)

Reports of included studies

(n = 86)

**Included**

* Duplicate records all removed by two reviewers

** All abstracts double screened using Rayyan by two blinded independent reviewers. All data double-extracted by two blinded independent reviewers. Conflicts were resolved by discussion; one involving a third reviewer (RK).

*** 12 studies were suitable for meta-analysis for an AI outcome as they contained both a CS and VB subsequent birth comparison group.

*From:*  Page MJ, McKenzie JE, Bossuyt PM, Boutron I, Hoffmann TC, Mulrow CD, et al. The PRISMA 2020 statement: an updated guideline for reporting systematic reviews. BMJ 2021;372:n71. doi: 10.1136/bmj.n71. For more information, visit: <http://www.prisma-statement.org/>
