# Supplementary material for: Caesarean section and anal incontinence in women after obstetric anal sphincter injury: A systematic review and meta‐analysis
Source: BJOG. 2024 Jul 4;132(8):1032–44. doi: 10.1111/1471-0528.17899 (PMC12137769; doi:10.1111/1471-0528.17899)
Supplement: Supplementary file 3 — Table S1. [file BJO-132-1032-s002.docx]

Table S1: Recurrence of OASI rates

| First Author, Year | Location | Setting/Type | Duration of study | Total OASIS in study period | Index OASI rate | Subsequent OASI incidence VB (n) | Subsequent OASI rate VB (%) |
| --- | --- | --- | --- | --- | --- | --- | --- |
| Abramowitz 2021 | Paris, France | Multicentre (5 teaching 1 DGH) Prospective RCT | 7y | Unknown | Unknown | 1 | **1.9% *** |
| Ali 2014 | Dublin, Ireland | Single centre, Teaching hospital, Retrospective review | **2y** | Unknown | Unknown | 11 | **13.40%** |
| Ampt 2015 | New South Wales | State Population Database, Retrospective review | 10y | 6380 | 4.5% | 276 | **5.70%** |
| An 2014 | Mercy Hospital, Australia | Single centre, Retrospective review | 4y | Unknown | Unknown | 1 | **2.70%** |
| Antonakou 2017 | UK | Single centre, DGH, Retrospective review | 9y | 603 | 5.40% | 16 | **8.40%** |
| Baghestan 2012 | Norway | National Population Database, Retrospective review | 37y | 21692 | 2.80% | 750 | **5.60%** |
| Basham 2014 | Chicago, USA | Single centre, Teaching hospital, Retrospective review | 5y | 1629 | Unknown | 23 | **3.20%** |
| Bayar 2013 | UK | Single centre, DGH, Retrospective review | 5y | Unknown | Unknown | 7 | **11.90%** |
| Bogeskov 2015 | Denmark | Multi centre (2) retrospective Case-Control Review | 11.5y | 2551 | 5.5% nulip, 1.5% multip | 49 | **8.00%** |
| Boggs 2014 | Canada (Ontario) | Single centre, Teaching hospital, State Population Database study | 4y | 9857 | 5.3% primip | 102 | **5.30%** |
| Dakin 2021 | Dublin, Ireland | Single centre, Teaching hospital, Retrospective review | 2y- follow up study (Ali 2014) | Unknown | Unknown | 4 | **4.3%** |
| Daly 2013 | Croydon UK | Single centre, Teaching hospital, Retrospective review | 10y | Unknown | Unknown | 19 | **7.60%** |
| Dandolu 2005 | Pennsylvania US | State Database study | 2y | 18888 | 7.31% | 864 | **5.76%** |
| Davies 2013 | Bristol, UK | Single centre, Teaching hospital, retrospective review | 6y | Unknown | Unknown | 201 | **10.2%** |
| De Souza 2020 | UK | Multi centre (4 Teaching hospitals) Retrospective Review | 12y | 4956 | 5.8% primip, 3.1% total | 180 | **10.20%** |
| Dilmaghani-Tabriz 2012 | UK | Single centre, DGH, Retrospective review | 3y | 139 | 3% | 1 | **6.66%** |
| Doumouchtsis 2014 | UK | Single centre, Teaching hospital, Retrospective review | 13y | 1702 | Unknown | 28 | **9.12%** |
| Edozien 2014 | UK | National Population Database, Retrospective review (HES data) | 7y |  | 3.80% | Unknown | **7.20%** |
| Edwards 2006 | Phildelphia USA | Single centre, Teaching hospital, Retrospective review | 13y | 778 | 3.3% | 6 | **2.4%** |
| Edwards 2019 | Michigan USA | Retrospective review, postal survey | 5m/5y | Unknown | Unknown | 2 | **4.90%** |
| Fitzpatrick 2016 | Dublin, Ireland | Single centre, Teaching hospital, Prospective Cohort | 7y | 1958 | Unknown | 20 | **5.10%** |
| Fradet-Menard 2018 | France | Single centre, Teaching hospital, Retrospective review (postal questionnaire) | 11y | 243 | 2% primip, 0.3% multip | 3 | **6.00%** |
| Harkin 2003 | Ireland | Single centre, Teaching hospital, Retrospective review | 3y | 342 | 1.70% | 2 | **4.40%** |
| Izzett 2015 | Kings, UK | Single centre, Teaching hospital, Retrospective review | 7y | 984 | 3.50% | 17 | **9.90%** |
| Jango 2016 | Denmark | National Population Database, Retrospective review, postal questionnaire | 9m/9y | 7336 | 4.60% | 521 | **7.11%** |
| Jordan 2018 | Croydon UK | Single centre, Teaching hospital, Prospective Cohort | 12y | Unknown | Unknown | 99 | **10.00%** |
| Lowder 2007 | Pittsburgh, Pennsylvania | Single centre, Teaching hospital, Retrospective review | 8y | 1054 | 16% primip, 3% multip | 76 | **7.20%** |
| Moffatt 2022 | Bristol UK | Single centre, Teaching hospital, Retrospective review | 1y | Unknown | 98 | 6 | **7.20%** |
| Nausheen 2014 | Mersey, UK | Single centre, Teaching hospital, Retrospective review | 6y | 188 | Unknown | 3 | **2.10%** |
| Nutaitis 2022 | USA | Single centre, Teaching hospital, Retrospective review | 2y | Unknown | Unknown | 3 | **1.90%** |
| Park 2015 | Sydney, Australia | Single centre, Teaching hospital, Retrospective review | 7y | 196 | 7.8% | 2 | **5.13%** |
| Parmar 2012 | USA | State Population Database, Retrospective review | 5y | 43583 | 11.60% | 2648 | **6.10%** |
| Pirhonen 2020 | Norway | Multinational Population Database, Retrospective review | 14y | 10327 | 4% (historic data used by authors) | 433 | **6.90%** |
|  | Sweden |  |  | 15270 |  | 480 | **4.50%** |
|  | Finland |  |  | 1001 | 1% (historic data used by authors) | 14 | **2.10%** |
| Ramalingam 2014 | Norway | Single centre, Teaching hospital, Retrospective review | 2y | 11635 | 2.1% | 1 | **11.1%** |
| Rahim 2021 | Imperial, UK | Single centre, Teaching hospital, Retrospective review | 5y | 642 | Unknown | 6 | **4.40%** |
| Spydslaug 2005 | Norway | National Population Database, Retrospective review | 32y | 9558 | 1.90% | 357 | **3.70%** |
| Tezschner 1996 | Denmark | University hospital | Unknown | 79 | 3.3% primip, 0.6% multip | 1 | **5.88%** |
| Thiel 2015 | Winchester, UK | DGH | 3y | Unknown | Unknown | 4 | **8.50%** |
| Van der Vlist 2020 | Netherlands | Single centre, Teaching Hospital, Retrospective review | 7y | 320 | 2.2% | 4 | **4.30%** |
| Van Bavel 2018 | Netherlands | National Population Database, Retrospective review | 10y | 9941 (first delivery) | 3.80% | Unknown | **5.80%** |
| Vasseur 2018 | Switzerland | Single centre, Teaching Hospital,  Retrospective Review | 9y | Unknown | Unknown | 2 | **3.60%** |
| Viner 2017 | Scotland | Single centre, DGH, Retrospective review | 2.5y | 23 | 1.20% | 4 | **25.00%** |
| Wegnelius 2011 | Stockholm | Single centre, Teaching Hospital, Retrospective review Case-Control study | 6y | Unknown | Unknown | 3 | **4.05%** |
| Woolner 2019 | Scotland | National Population Database, Retrospective review | 14y | 5174 | 2.80% | 149 | **6.60%** |
| Yogev 2014 | Tel Aviv, Israel | Single centre Teaching hospital | 13y | 356 | 0.60% | 4 | **1.96%** |
| Young 2022 | Australia | Single centre, Teaching Hospital, Retrospective review | 7y | 828 | 3.03% | 9 | **5.40%** |
| Yousif 2011 | Ireland | Single centre, Retrospective review | 10m | Unknown | Unknown | 1 | **12.50%** |

* as not every woman had a 3rd/4^th^ degree in 1^st^ delivery this may not represent a recurrent OASI
