# Supplementary material for: Caesarean section and anal incontinence in women after obstetric anal sphincter injury: A systematic review and meta‐analysis
Source: BJOG. 2024 Jul 4;132(8):1032–44. doi: 10.1111/1471-0528.17899 (PMC12137769; doi:10.1111/1471-0528.17899)

Anal incontinence (AI) A: solid, B: liquid, C: flatal and D: fecal urgency, all time periods.

A: Solid

Study or Subgroup	CS Events	CS Total	VB Events	VB Total	Weight	Odds Ratio M-H, Fixed, 95% CI
Bogeskov 2015	0	26	3	27	13.8%	0.13 [0.01, 2.69]
Jango 2016	11	506	42	1472	86.2%	0.76 [0.39, 1.48]
Jordan 2018	0	23	0	99		Not estimable
**Total (95% CI)**		**555**		**1598**	**100.0%**	**0.67 [0.35, 1.29]**
Total events	11		45			

Heterogeneity: Chi² = 1.24, df = 1 (P = 0.27); I² = 19%
Test for overall effect: Z = 1.20 (P = 0.23)

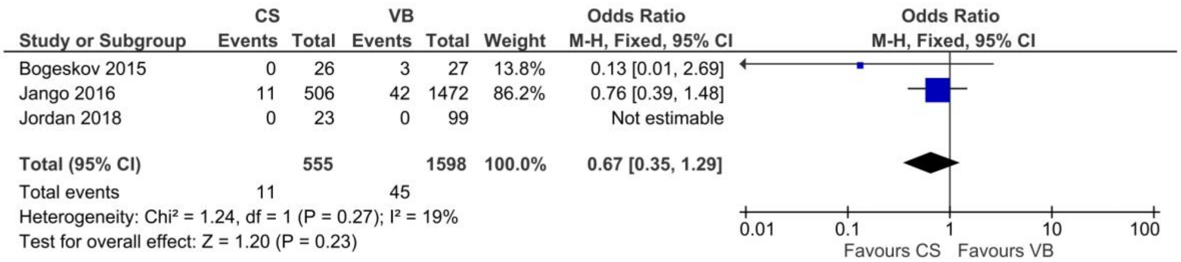

B: Liquid

Study or Subgroup	CS Events	CS Total	VB Events	VB Total	Weight	Odds Ratio M-H, Fixed, 95% CI
Bogeskov 2015	0	26	1	27	3.7%	0.33 [0.01, 8.56]
Jango 2016	17	506	73	1472	92.6%	0.67 [0.39, 1.14]
Jordan 2018	1	23	4	99	3.7%	1.08 [0.11, 10.14]
**Total (95% CI)**		**555**		**1598**	**100.0%**	**0.67 [0.40, 1.12]**
Total events	18		78			

Heterogeneity: Chi² = 0.35, df = 2 (P = 0.84); I² = 0%
Test for overall effect: Z = 1.52 (P = 0.13)

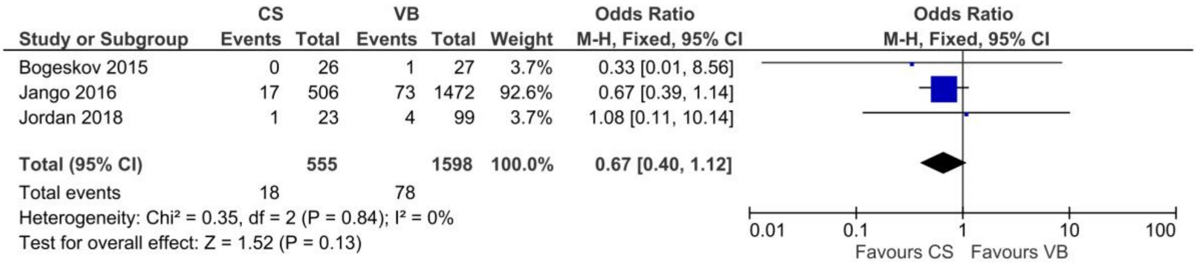

C: Flatal

Study or Subgroup	CS Events	CS Total	VB Events	VB Total	Weight	Odds Ratio M-H, Fixed, 95% CI
Bogeskov 2015	9	26	8	27	4.1%	1.26 [0.40, 3.99]
Dilmaghani-Tabriz 2012	0	15	2	13	2.1%	0.15 [0.01, 3.40]
Jango 2016	103	506	259	1472	83.8%	1.20 [0.93, 1.54]
Jordan 2018	3	23	7	99	1.8%	1.97 [0.47, 8.29]
Webb 2020	8	51	15	74	8.2%	0.73 [0.28, 1.88]
**Total (95% CI)**		**621**		**1685**	**100.0%**	**1.15 [0.91, 1.46]**
Total events	123		291			

Heterogeneity: Chi² = 3.18, df = 4 (P = 0.53); I² = 0%
Test for overall effect: Z = 1.19 (P = 0.23)

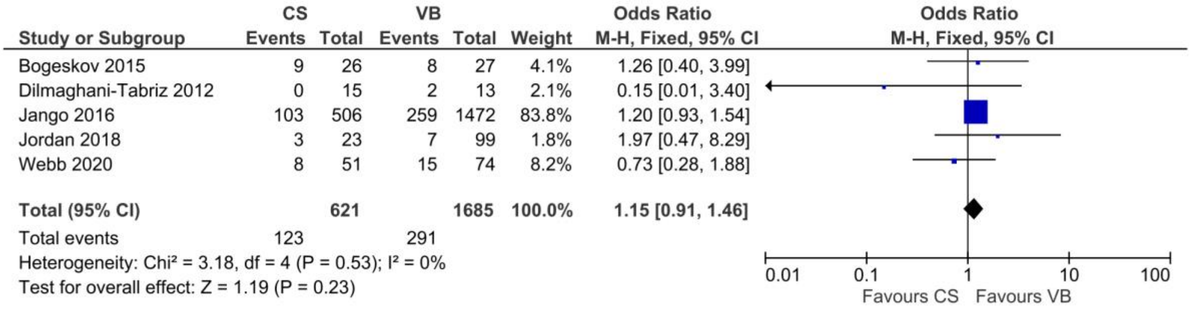

D: Urgency

Study or Subgroup	CS Events	CS Total	VB Events	VB Total	Weight	Odds Ratio M-H, Random, 95% CI
Bogeskov 2015	2	26	1	27	8.2%	2.17 [0.18, 25.46]
Jango 2016	181	506	407	1472	49.9%	1.46 [1.18, 1.81]
Jordan 2018	0	23	8	99	6.3%	0.23 [0.01, 4.11]
Karmarkar 2015	2	24	0	26	5.6%	5.89 [0.27, 129.15]
Webb 2020	8	51	20	74	30.0%	0.50 [0.20, 1.25]
**Total (95% CI)**		**630**		**1698**	**100.0%**	**1.05 [0.49, 2.27]**
Total events	193		436			

Heterogeneity: Tau² = 0.30; Chi² = 7.44, df = 4 (P = 0.11); I² = 46%
Test for overall effect: Z = 0.13 (P = 0.90)

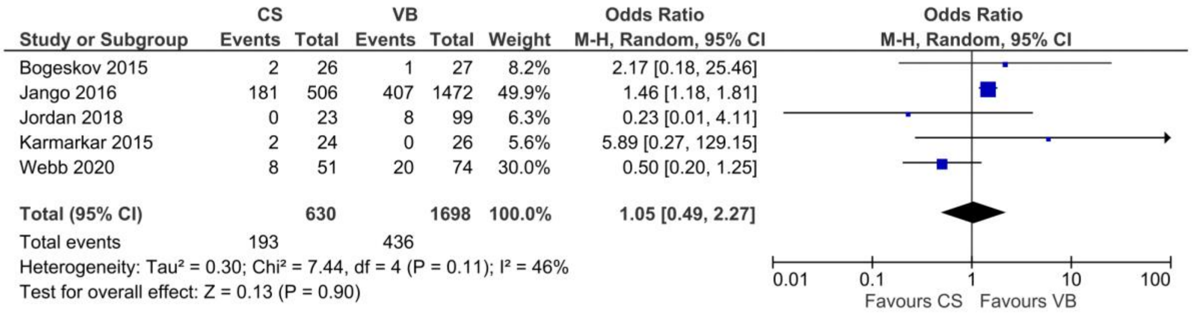

Supplement: Supplementary file 4 — Figure S2. [file BJO-132-1032-s003.pdf]
