# Supplementary material for: Caesarean section and anal incontinence in women after obstetric anal sphincter injury: A systematic review and meta‐analysis
Source: BJOG. 2024 Jul 4;132(8):1032–44. doi: 10.1111/1471-0528.17899 (PMC12137769; doi:10.1111/1471-0528.17899)

Quality of life, satisfaction and regret, all time periods

A: Quality of life

Study or Subgroup	CS Events	CS Total	VB Events	VB Total	Weight	Odds Ratio M-H, Random, 95% CI
Jango 2016	210	506	365	1472	56.3%	2.15 [1.74, 2.66]
Webb 2020	5	51	14	74	43.7%	0.47 [0.16, 1.39]
**Total (95% CI)**		**557**		**1546**	**100.0%**	**1.10 [0.25, 4.89]**
Total events	215		379			

Heterogeneity: Tau² = 1.01; Chi² = 7.30, df = 1 (P = 0.007); I² = 86%
Test for overall effect: Z = 0.13 (P = 0.90)

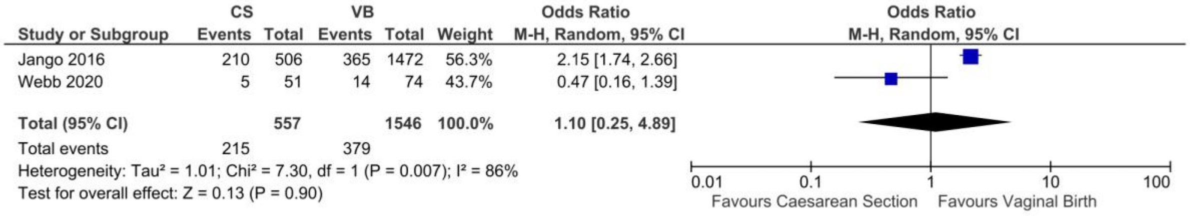

B: Satisfaction with mode of subsequent birth

Study or Subgroup	CS Events	CS Total	VB Events	VB Total	Weight	Odds Ratio M-H, Fixed, 95% CI
Fitzpatrick 2016	58	58	135	139	12.0%	3.89 [0.21, 73.34]
Young 2022	22	27	44	61	88.0%	1.70 [0.55, 5.21]
**Total (95% CI)**		**85**		**200**	**100.0%**	**1.96 [0.70, 5.53]**
Total events	80		179			

Heterogeneity: Chi² = 0.27, df = 1 (P = 0.60); I² = 0%
Test for overall effect: Z = 1.27 (P = 0.20)

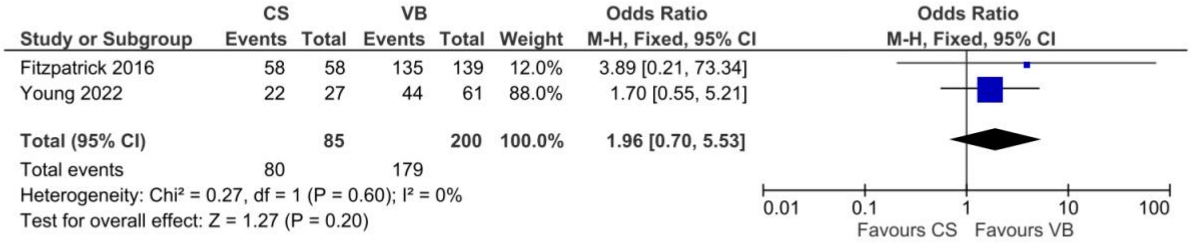

C: Regret with mode of subsequent birth

Study or Subgroup	CS Events	CS Total	VB Events	VB Total	Weight	Odds Ratio M-H, Fixed, 95% CI
Bogeskov 2015	2	9	11	47	60.5%	0.94 [0.17, 5.17]
Edwards 2019	0	8	5	42	39.5%	0.40 [0.02, 7.97]
**Total (95% CI)**		**17**		**89**	**100.0%**	**0.72 [0.17, 3.12]**
Total events	2		16			

Heterogeneity: Chi² = 0.24, df = 1 (P = 0.63); I² = 0%
Test for overall effect: Z = 0.43 (P = 0.66)

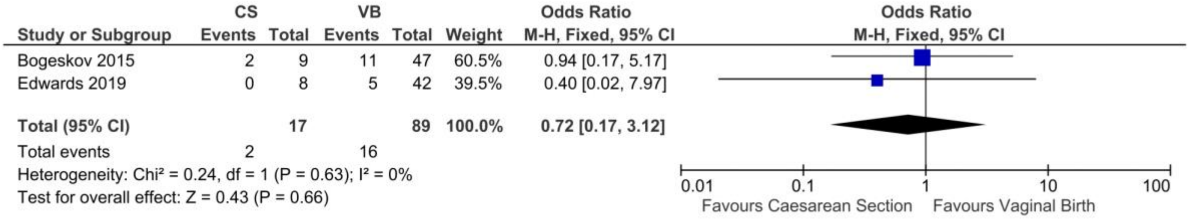

Supplement: Supplementary file 5 — Figure S3. [file BJO-132-1032-s001.pdf]
